# Ten simple rules for organizing a webinar series

**DOI:** 10.1371/journal.pcbi.1006671

**Published:** 2019-04-01

**Authors:** Faisal M. Fadlelmola, Sumir Panji, Azza E. Ahmed, Amel Ghouila, Wisdom A. Akurugu, Jean-Baka Domelevo Entfellner, Oussema Souiai, Nicola Mulder, H3ABioNet Research working group as members of the H3Africa Consortium

**Affiliations:** 1 Centre for Bioinformatics and Systems Biology, Faculty of Science, University of Khartoum, Khartoum, Sudan; 2 Computational Biology Division, Department of Integrative Biomedical Sciences, University of Cape Town, Cape Town, South Africa; 3 Department of Electrical and Electronic Engineering, Faculty of Engineering, University of Khartoum, Khartoum, Sudan; 4 Laboratory of Transmission, Control and Immunobiology of Infections (LTCII), Institut Pasteur de Tunis, Tunis-Belvédère, Tunisia; 5 Noguchi Memorial Institute for Medical Research, University of Ghana, Legon, Accra, Ghana; 6 South African MRC Bioinformatics Unit, South African National Bioinformatics Institute, University of the Western Cape, Bellville 7535, Cape Town, South Africa; 7 Laboratory of BioInformatics Biomathematics and bioStatistics, Institut Pasteur de SalTunis, Tunis, Tunisia; 8 Institut supérieur des technologies médicales, Univesité Tunis al Manar, Tunis, Tunisia; Dassault Systemes BIOVIA, UNITED STATES

## Introduction

Technological advancements are rapidly changing the face of science in terms of data acquisition, its transfer, storage, analysis, interpretation and dissemination of results [1]. In biology and genomics, this is affecting many traditionally considered purely wet lab experiments like genome sequencing [2], medical diagnosis [3], and drug design [4]. It therefore becomes essential for bioinformaticians to remain up to date with recent trends and innovations in the field. Within Africa, this is even more true as the continent is striving to foster the development of innovative tools and strategies to improve health outcomes on the continent. H3ABioNet [5], the pan-African Bioinformatics Network, was established with the aim of capacity building in mind to further advance genomics research in Africa. It is therefore complementary to its other training initiatives [6] to ensure African scientists have access to avenues to disseminate their research, discuss their work, and network with peers.

Seminars and conferences are good opportunities for sharing and discussing new insights and networking with peers and can be considered as scientific meetings [7–13]. However, with prohibitive traveling costs and increased logistics, it is not always feasible to organize and attend numerous regular seminars. In an increasingly interconnected world brought about by technological advancements in communications, other alternatives can be used to supplement the in-person experience. Examples within Africa include: the H3ABioNet offering of a hybrid-delivery 3-months course, Introduction to Bioinformatics [18]. The Global Women in Data Science (WiDS) conference is another example of a one-day technical conference that is live-streamed from various locations across the globe (http://www.widsconference.org/). Mozilla Open Leaders (https://mozilla.github.io/leadership-training/), and global sprints (https://foundation.mozilla.org/en/opportunity/global-sprint/) are other examples of active engagement and community building that are arranged and conducted remotely.

Through organizing regular online seminars, known as webinars, H3ABioNet is aiming to empower a predominantly African audience to reap the benefits of being kept abreast of current research trends by expert domain scientists.

Webinars present a great virtual opportunity to engage and stimulate interactions between presenters and participants, and can accommodate more participants than a physical conference room setting which could be limited by space and accessibility [8]. Webinars provide participants the convenience of attending an academic presentation from the comfort of their offices or homes while multi-tasking. A successful webinar session is strongly dependent on the planning activities prior to the session.

We share in this paper ten simple rules for hosting a regular webinar series with particular emphasis on resource-constrained communities like many in Africa. These rules are derived from experiences gained and lessons learnt while organizing and running the H3ABioNet bioinformatics webinar series.

## Rule 1: Assemble an effective webinar coordination team

Similar to organizing any scientific meeting [7], hosting of a regular webinar series requires the involvement of a dedicated group of people. The role of the webinar coordination team is to assist with all the planning and logistics for hosting a webinar, while ensuring that the workload is not borne solely by a single individual. The involvement of postgraduate students and postdocs as part of the webinar coordination team facilitates the development of their skills in planning, communication, use of various conferencing platforms, coordinating and hosting of scientific events. These soft skills are vital for collaboration and working in large consortia which are not usually part of their normal training. To ensure the smooth running of the webinars, regular planning and post webinar meetings play an important role in iteratively developing and refining the planning procedures based on the challenges and successes of the previous webinars (Rule 10). This helps develop a cohesive and cooperative team of coordinators despite the fact that they might be located in different time zones and with busy schedules.

## Rule 2: Align a webinar theme to the expectations of the audience

Webinars are used as platforms to enable knowledge exchange, and to disseminate methods, results and best practices. They are generally aimed at specific audiences and address specific themes. Thus, choosing webinar themes requires mapping of the target audience needs and interests [7].

A webinar series could be instrumental in capacity building in various ways: 1) By hosting early career researchers, which gives them an opportunity to get early feedback on their research findings and practice scientific presentations skills. Given their limited experience, shorter durations (around 20 minutes talk each) are appropriate. 2) By hosting senior researchers in areas of interest to the intended audience. One senior scientist per session that lasts for 45-50 minutes are more reasonable. This enables the early career and junior researchers to expand their horizons and polish their ideas. It may also open avenues for collaboration or spring new research directions.

## Rule 3: Consider a webinar planning checklist

**Fig 1:**
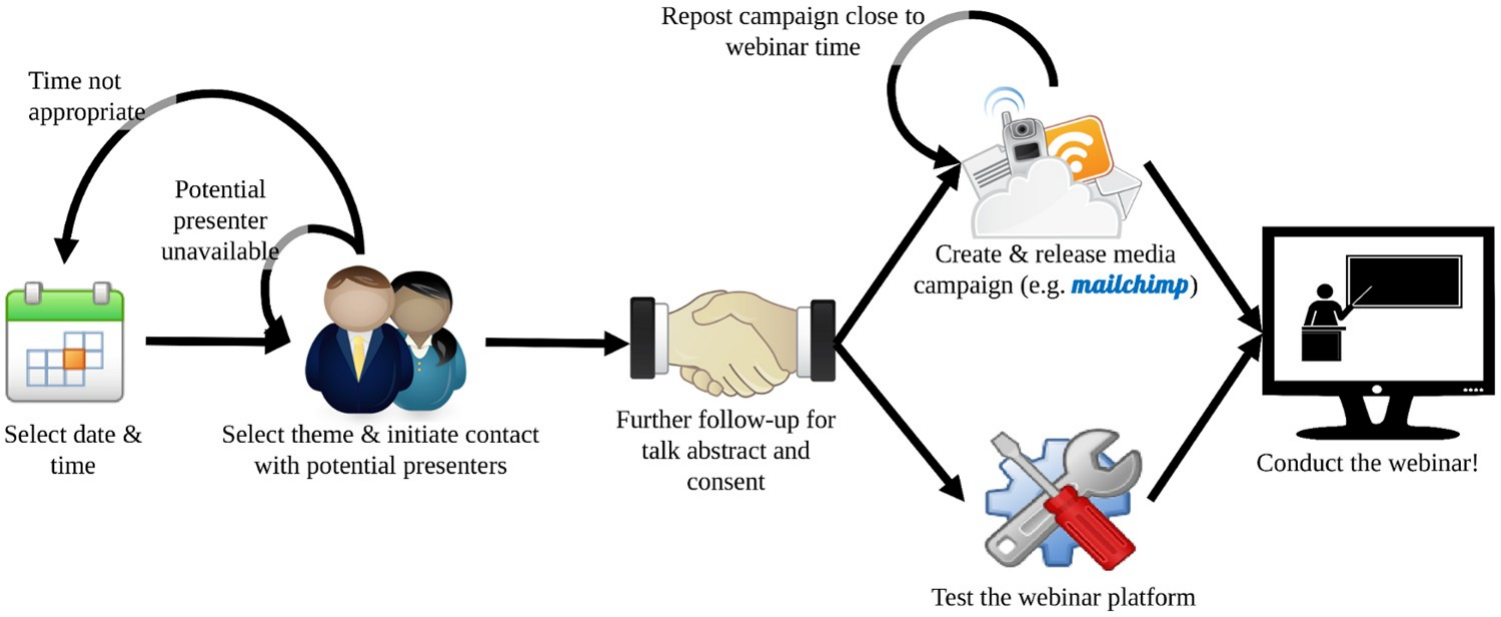
Planning flowchart. A webinar series flowchart of planning and logistics activities.

The main pre-webinar planning objectives and activities should be drafted and agreed upon by the webinar coordination team and a recurring list of tasks and responsibilities should be identified and drafted from the outset so each person’s roles and responsibilities are clear (see Fig 1). It is important to include all the webinar team members in the identification and planning of the tasks from the start so they are more engaged as stakeholders and facilitate task distribution. The earlier tasks and timelines are identified, the better in terms of time available to prepare for the hosting of a webinar. Tasks include: determining the date for the webinar (rule 5), approaching potential speakers, obtaining the webinar talk, abstract and presenter’s biography for creating the webinar announcement (rule 8), having a test run with the presenter (rule 9) using the chosen webinar platform (rule 6).

## Rule 4: Share webinar organizational documents

An accessible shared space for webinar documents enables decentralized access and smooth organization of tasks and resources. Various template documents on organizational letterheads can be created as the basis for gathering information from invited webinar speakers. Information collected should include the webinar title, authors, abstract, a picture of the speaker and their biography. An important document required is a consent release form whereby the speakers give their permission to use their submitted picture for the webinar announcement, record the webinar and acknowledge that they are the authors of the work. The webinar consent form also asks the speaker to choose which Creative Commons (CC) license they would like to link to the talk (CC by SA is the recommended license) and most importantly provides the speaker’s permission to host the recorded webinar on various platforms such as a website or YouTube channel. The completion of these templates also allows the webinar coordinators to check with the speakers if there might be any sensitive unpublished results in the talk and discuss whether they should be presented. Various information captured through these templates are used to generate a webinar announcement (rule 8). For consistency, the same announcement format is used for all the webinars.

## Rule 5: Plan early and devise a calendar of regular activities

The regular cycle of hosting regularly occurring webinars inevitably translates into a series of recurring deadlines. Creating a calendar of webinar events helps mitigate the sudden onset of deadlines. In doing so, it is sufficient to settle on the provisional dates and times of the webinar events along with their themes. Speakers can be identified and confirmed later on accordingly (see Rule 7). It’s important to pick the webinars time to accommodate audiences in various time zones. In today’s increasingly interconnected world, there will inevitably be clashes with other meetings or workshops. Hence the earlier a regular webinar date and time is settled upon, the earlier it will make its way into attendees’ calendars thereby enabling them to avoid subsequent scheduling conflicts. Another advantage of devising a calendar of activities earlier, is that due dates with reminders to perform specific tasks can be set up well in advance, themes settled upon and potential speakers contacted reasonably early enough for their availability to provide a talk before their calendars are filled.

## Rule 6: Settle on a convenient and user friendly webinar platform

The choice of platform for hosting a webinar is crucial. There are numerous free and paid-for platforms available offering different features. An important point to take into consideration within resource-limited settings is that most regions are operating on very constrained bandwidth and have limited budgets, so the use of expensive webinar platforms that have high bandwidth requirements and charge per user may not be feasible.

Free and commercial platforms exist that may be suitable for streaming webinars and other interactive Online events, such as: GoToMeeting, Zoom, Adobe Connect, Vidyo, Mconf and Google hangouts. These platforms vary in the features they provide, and it is of value to compare and assess before committing to a platform. Table 1 provides an example comparison of the key features offered in two platforms evaluated by the authors.

**Table 1:** Comparison of the two webinar platforms used.

Comparison aspect	Google Hangouts	Mconf
Free/open source	Free	License
Ease of use	Could require Hangouts plugin installed; works best with Chrome browser	Requires Flash installed, works best with Mozilla Firefox browser.
Video & Screen sharing	Yes	Yes
Recorded videos	Readily available as Youtube videos	Available as html page files that requires rendering
Room size limit	10 (free plan)—25 (business & education)	50-60
Privacy	Can join from Hangouts (with Gmail account), or from Youtube (Anonymously)	Login with anonymous guest name or registered mconf user
Notable problems	“Trying to reconnect” error	All traffic goes through central servers, that can be occasionally overloaded or down.

## Rule 7: Select theme expert presenters

The success of a good webinar hinges on the relevance of the talk to the proposed theme. As the various themes have been decided beforehand, the webinar coordination team members are able to reach out to other consortium members to identify speakers with relevant domain expertise. An advantage of having predetermined themes for a webinar is the flexibility to pre-identify more than one potential domain expert speaker.

In case of non-availability of the first potential speaker approached, other identified speakers can be approached. One of the webinar coordination team members drafts an invitation message to the potential presenter which includes the date and time of the webinar. Once an invited webinar speaker accepts the invitation to present, the template to capture the webinar abstract and the speaker’s, biography and the webinar recording consent form are sent to the presenter with a due date for completion. A request is also made to provide at least three convenient dates and time slots to run through the logistics of the webinar platform being used and the format of the webinar.

Inclusion of all the coordination team members during communication with invited speakers will serve as a backup for any follow-up responses.

## Rule 8: Announce webinars through mailing lists and social media platform

Key to attracting a diverse and active audience is the dissemination of the webinar announcement earlier, and through both relevant mailing lists and social media channels that the target audience typically use (rule 2). The webinar announcements, containing all the required information: date, time, tool or web address, talk title, abstract and speaker’s biography can be designed in marketing platforms like MailChimp for effective dissemination. Social sharing icons on the webinar announcement, use of relevant hashtags, and mentions of the speakers and their institutes all contribute to increased visibility of a given webinar announcement.

Twitter is one of the most used social channels for live webinars. You might consider running a live Q&A on Twitter in conjunction with your webinar. This can be done through designated hashtag or by posting questions to your audience and asking them to reply. It’s good to see what your audience is saying to you and each other about your presentation. During the event, your moderator or social media coordinator should be highly visible on Twitter: answering questions, sharing interesting stats, engaging with attendees, and routing technical challenges.

## Rule 9: Allocate time for the platform orientation

Testing of the platform with the presenter is crucial to ensure a successful webinar session. This includes going through the webinar interface with the speaker to familiarise her/him by navigating through the presentation slides and testing different functionalities such as a mouse guided pointer.

The webinar flow is also discussed with the speaker during the test. Pre-testing also enables the webinar coordination team to assist the speaker fix any potential software or dependency issues before the actual webinar. Some platforms require initial add-ons or software setup which the presenter might not have installed. Depending on the platform used, sufficient details and instructions on how to test connection and/or software should be provided in the webinar announcement, usually in the form of web address to test the connection.

## Rule 10: Iteratively assess and evaluate what works and what doesn’t

Running and maintaining scientific events like a recurring webinar series needs keeping careful track of metrics for regular assessment and evaluation [14]. Keep in mind that the online environment is still an evolving and untested media, and you will undoubtedly have to adopt a trial-and-error approach to find what works best [15].

In order to assess the webinars, a post webinar survey should be shared with all attendees. Such a survey could also ask for suggestions on themes or topics to be included in the webinar series.

Most of the webinar audience will normally go through the webinar advert and based on the advertised themes decide to attend a particular session. When such expectations are not met, webinars tend to have low participation. Webinars with pictures, graphs, tables and other diagrammatic representation get participants much more interested in following the session to the end than textual presentations [16, 17]. Webinars’ presenters need to carefully develop a presentation that truly reflects the advertised themes while devising ways to sustain the audience throughout the session. More efforts in the training of early career researchers on quality and professional presentation is highly recommended.

## Summary

The webinars series form part of the regular activities of any scientific consortium that aims to strengthen research activities and foster collaborations amongst the different partners. The webinars are intended to foster the exchange of ideas, build potential collaborations across multiple disciplines and enabling the participation and sharing of knowledge in current research.

The Ten Simple Rules for organizing a webinar series can be summarized as follow: The webinar coordination team assists with all the planning and logistics for hosting a webinar (Rule 1); Choosing webinar themes requires mapping of the target audience needs and interest (Rule 2); Drafting a webinar planning checklist through regular planning meetings as well as post webinar meetings (Rule 3); Decentralized webinar organization of tasks and resources through accessible shared space (Rule 4); Planing early and settling on the provisional dates and times of the webinar events along with their themes (Rule 5); Choosing and settling on convenient and user friendly webinar platform (Rule 6); Approaching and confirming potential speakers (Rule 7); Obtaining the webinar title, abstract and presenter’s biography for creating the webinar announcement through emails and social media channels (Rule 8); Allocating time for the platform orientation (Rule 9); and Keeping close-up track of webinar metrics for regular assessment and evaluation (Rule 10).

These Ten Simple Rules shared with the computational biology community will help those who have not yet ventured into training through webinars to learn from our experience. In our experience, the feedback from the post-webinar surveys clearly demonstrate that webinars are an effective way to create a two-way conversation between presenters and participants via a web-based platform.
