# Notice of Republication: Ten simple rules for organizing a webinar series

**DOI:** 10.1371/journal.pcbi.1007048

**Published:** 2019-05-08

**Authors:** The PLOS Computational Biology Staff

This article was republished on April 11^th^, 2019, to correct an error in the name of an author that affected the citation, and to add the ORCiD for the author Azza E. Ahmed that was accidentally missed. The publisher apologizes for the error.

## Supporting information

S1 File Originally published, uncorrected article. (PDF)

S2 File Republished corrected article. (PDF)

## References

[1] Fadlelmola FM, Panji S, Ahmed AE, Ghouila A, Akurugu WA, Domelevo Entfellner J-B, et al (2019) Ten simple rules for organizing a webinar series. PLoS Comput Biol 15(4): e1006671 10.1371/journal.pcbi.1006671 30933972 PMC6443143

